# Intermarriage: Or, the Mode in Which, and the Causes Why, Beauty, Health, and Intellect Result from Certain Unions; and Deformity, Disease, and Insanity from Others, &c.

**Published:** 1839-04

**Authors:** 


### Art. III.

*Intermarriage: or, the Mode in which, and the Causes why, Beauty, Health, and Intellect result from certain Unions; and Deformity, Disease, and Insanity from others; demonstrated by Delineations of the Structure and Forms, and Descriptions of the Functions and Capacities which each Parent, in every Pair, bestows on Children,—in conformity with certain Natural Laws, and by an Account of corresponding Effects in the Breeding of Animals. Illustrated by Drawings of Parents and Progeny.* By Alexander Walker.—*London*, 1838. 8vo. pp. 442.

This is in many respects a very remarkable book. We are not prepared to admit that its author has fulfilled all the expectations excited by his title-page; nor are we disposed to go the whole length with him in the positions he maintains: but he has collected in support of them a mass of facts, many of them as novel as they are unimpeachable, which renders his volume alike important and interesting to the physiologist. In attempting to adapt it to the taste and capacity of the public, however, the author seems to us to have been led into the common error of introducing a quantity of comparatively worthless matter, which has a somewhat repulsive character to the scientific enquirer. We believe that there is scarcely one of the higher truths of any science, whether physical or vital, which may not, by proper explanation and illustration, be brought home to the comprehension of any person of ordinary intelligence; but we cannot think the addition of a heap of common-place trifles, calculated only to attract the curiosity of the vulgar, a labour worthy of a man of science. We certainly see no reason why the public mind should be less instructed upon such subjects than upon any other department of physiology. But all depends upon the *mode* in which the instruction is given; and whilst, on the one hand, we know, from experience, that the fundamental truths relating to the function of reproduction, both in vegetables and in animals, may be expounded in such a manner as not to raise a blush on the cheek of one sex or to excite an impure feeling in well-regulated minds in the other, we also know that, in too many of the works designed for general circulation, the converse is too apparent; all things becoming impure to the man of gross and sensual imagination. We do not mean to charge Mr. Walker, however, with the commission of this fault to an extent at all to be compared with that of which we, a short time since, expressed the strongest reprobation; but we must say that, if there had been less in his work of an *ad captandum* nature, we should have had much more satisfaction in recommending it to the general reader, as we have already done to the physiological enquirer.

Mr. Walker has very properly dedicated his work to Mr. Knight, whose extensive researches on a corresponding department of vegetable physiology are well known, and who has contributed many of the most valuable observations contained in the present volume. The recent loss of this eminent and highly respected individual has deprived science of one of her most assiduous votaries; and his last contribution to the Transactions of the Royal Society (which will presently be noticed) is an ample proof that the zealous devotion of a long life to philosophical pursuits need cause no diminution in the relish for them, even in declining years.

In an advertisement, or rather preface, the author explains, at more length than in his title-page, the plan and objects of his treatise; and these we shall set before our readers in his own words, but somewhat abbreviated.

"The great object of this work is altogether new, and heretofore unattempted,—the establishment not merely of a new science, but of that science which is by far the most interesting to humanity, the science which, for the first time, points out and explains all the natural laws that, according to each particular choice in intermarriage, determine the precise forms and qualities of the progeny; which unfolds the mode in which and the causes why beauty, health, and intellect result from certain unions, and deformity, disease, and insanity from others; and which enables us, under all given conditions, and with absolute certainty, to predict the degree and kind of these which must result from each intermarriage.

"The philosophical bases of this science have, moreover, nothing to do with hypothesis or supposition; they are the indisputable though hitherto unapplied facts of anatomy and physiology; and their present popular applications are rendered subjects of absolute demonstration by descriptions and drawings of families, (some of them well known to the public;) while every reader has the power of adding to their number among the families of his acquaintance. They are further subjected to demonstration by all the more important facts here stated, as to the breeding of domesticated animals; facts which have not hitherto been explained or understood, and consequently have not hitherto afforded those principles on which the breeder may *now* act, with perfect certainty of the desired result.

"In the First Part of the work is given an account of the physiological conditions connected with and terminating in LOVE; the period of puberty, and the remarkable and interesting changes which it causes in the locomotive system and the voice, in the vital or nutritive system, and in the mental or thinking system, especially of woman. . . .

"In the Second Part are described the sexual relations arising from these conditions, and connected with or leading to INTERMARRIAGE. . . .

"In the Third Part are described the circumstances resulting from the preceding relations, and connected with or productive of PROGENY. . . .

"In the Fourth Part are enunciated the newly discovered laws regulating the RESEMBLANCE OF PROGENY TO PARENTS; the law of selection, where both parents are of the same variety; the law of crossing, where each parent is of a different variety; the law of in-and-in breeding, where both parents are of the same family; the law of sex, &c. . . .

"In the Fifth and Sixth Parts are described the vague methods of regulating progeny adopted in the breeding of Domesticated Animals. . . .

"In the Seventh and Eighth Parts are described the vague methods of affecting progeny adopted among MANKIND; in in-and-in, selection, and crossing; and the transcendently important subject of choice in intermarriage." (p. xvii.)

How far the author has accomplished all which he has attempted we shall now examine, passing over with slight notice the First and Second Parts, which contain a good deal that, in our judgment, is not only extraneous but offensive. We cannot say that we should have discovered anything peculiarly original in this portion of Mr. Walker's treatise, (which seems to us principally derived from the works of Virey and other prurient French writers,) had it not been for the claims advanced by the author himself. At the close of the advertisement from which we have already quoted, we find a list of sixty-one propositions, embracing "the more important original facts and opinions which the work contains." Among these is specified (No. 2) "the assignments of the cause of early puberty and of the catamenia in woman." That our readers may not be left in ignorance of this notable discovery, we shall give it in the author's own words:

"In early life, the three classes of organs and functions—the locomotive, the vital or nutritive, and the mental or thinking systems,—bear the same proportion to each other in woman as in man; and the girl is scarcely distinguishable from the boy. In woman, this proportion is gradually departed from: her vital system, occupying chiefly the trunk, becomes larger in general as well as in particular parts; it grows out of proportion to the other two systems, occupying chiefly the head, or composing the limbs; its functions follow its structure; and hence alone the earliness of that aggregate of them which is denominated puberty." (p. 7.)

This seems to us to imply nothing more or less than the systems of organic life are altogether in a state of more advanced development in the female than the male, after the period of early childhood; a fact which has long been observed, and which has been curiously confirmed as to *weight* by the statistical researches of Quetelet, who found that, whilst the weight of the male predominates up to the age of eight or ten years, the increase between that period and the age of twelve years is so rapid that, at the latter epoch, the average weight of the female is equal to that of the male. Subsequently, however, the male gains again upon the female, and continues to increase both in weight and height after she has attained her full adult period: an increase in weight, however, takes place in woman after the termination of the parturient period, but this evidently results from other causes. By a collection of facts of this kind, we think that Mr. Walker might have made his book much more valuable, and not less interesting to the general reader. Another circumstance occurs to us as curiously illustrative of the resemblance which the children of both sexes bear to each other and to the female parent. When the plumage of male birds (as usually happens) is more brilliant than that of the female, the young birds of both sexes resemble the latter in their early dress; just as among the part of mankind accustomed to the artificial refinements of clothing, little boys as well as girls are made to wear petticoats before being advanced to the dignity of breeches.

As to the explanation of the cause of the catamenial discharge, we cannot afford to Mr. Walker the merit which he claims for himself; and we think it would have been much wiser in him to have rested his title to distinction on the doctrines, of which the originality, to say the least, no one can dispute. But our readers shall again judge for themselves.

"Woman is every month subject to a sanguineous flow from the matrix; an universal and essential event in the life of the female. The cause of this is evidently the same with that of her early puberty, the disproportion in which the vital system is to the locomotive and nervous systems. Thus, the female becomes possessed of a greater quantity of blood than is required for her individual preservation. Thus, she is enabled, when pregnant, to supply a sufficient quantity for the nourishment of the fœtus. Thus, when suckling, she can afford the vast secretion of milk; and thus, at all other periods, this blood, being voided, furnishes the catamenial flow." (p. 36.)

Now this explanation, which has been given by fifty authors at least, in different language, is liable to this prominent objection, that the menstrual fluid is *not* blood, but is essentially a secretion, being deficient in one of the most important constituents of blood, namely, the fibrin. That this periodical secretion is necessary to the preservation of the healthy condition of the generative organs, as well as of the system at large, everybody knows; but that it does so merely by letting out the superabundant amount of blood, we deem a very false doctrine, since the ingredient which seems most essential to the formation and nutrition of solid tissues is not parted with in this manner.

From the predominance of the vital system in females, Mr. W. jumps to the conclusion that the pleasures of love are more exquisitely enjoyed by them than by the other sex; and thus solves the question which has been so frequently asked, from the time of Tiresias downwards. We shall not enter into a grave discussion of this knotty question, but simply point out that here, as in other parts of his work, Mr. Walker seems to have forgotten that distinction between the instinctive propensity which results only from the excitement of the genital system, and the moral sentiment which may exist independently of it, and which is the peculiar characteristic of the attachment between the sexes in the human species.

The Third Part of Mr. Walker's work contains much that is interesting and valuable to the physiologist. It is an old observation, that sexual attachments, both in man and the lower animals, are the strongest where each individual possesses, in the highest degree, the characteristics of the respective sex. Mr. Walker has, in his explanation of the "natural preference for various kinds of beauty," shown this principle of difference to have an extensive application. The following extracts contain his ideas upon this subject.

"There is a positive and a relative beauty: in other words, beauty differs not only in the two sexes, and in every individual of each sex, but each individual forms a different estimate of it in relation to himself. Hence, while he confesses the supremacy of a general model of beauty, and grants the superiority of the woman who most nearly approaches to it, he, for himself, decides in favour of another woman, whose beauty is less regular but more suitable to his desires. This curious fact has been often noticed, but never explained." (p. 107.)

"In my work entitled 'Beauty illustrated chiefly by an Analysis and Classification of Beauty in Woman,' it has been shown that, though one particular species of beauty will be found at all times to predominate in each individual woman, yet that there is ever a tendency, in the young woman, to beauty of the locomotive system; in the middle-aged woman, to beauty of the vital or nutritive system; and, in the older woman, to beauty of the mental or thinking system. It is not less remarkable that men of various ages generally admire precisely those species of beauty which prevail in women at corresponding ages." (p. 113.)

"As, however, woman is more precocious than man, she becomes more advanced, in reference to sex, than man at the same age; and, consequently, to be duly matched to her husband, the wife should be the younger.

"Of this admiration, then, and the consequent preference, modified as it is by age, it is necessary that the foundation should be explained. That foundation appears to be the similarity of objects and interests which are inseparable from similar periods of life, the association of these with a similar intensity of sexual desire, the consequent production of similar sympathy, and the resolve that it shall be permanent. This admiration and preference of corresponding ages secure in their turn those objects and interests without which there could be no happy superstructure." . . "Suitable states of the vital system happily accompany this sympathy, admiration, and preference as to ages." . . .

"It would appear, then, that sympathy, admiration, and preference being thus formed, each sex naturally and necessarily seeks next, not for qualities which are its own, but for those of which it is not in possession. It seeks not those, however, in other species, where not only due adaptation for sexual purposes, but all relations of sympathy are wanting. It seeks them the less even in the varieties of its species, that such adaptation and relation are very defective, as will be shown in the sequel. No being, then, can desire that of which it is already in possession; and the preference of that which is different from itself is founded on the absolute necessity of difference to all excitement. An animal cannot feel sexual excitement towards itself; it can feel little towards that which is like itself; it must feel most toward that which is most unlike it." (pp. 115, 116.)

In illustration and support of this view, the preference usually observed on the part of man for females of inferior height, and on the part of women for men of loftier stature; the dislike felt by women for men of effeminate character, and by men for women of masculine temperament; and the opposite results arising from a reversion of the usual conditions, are, we think, justly adduced by our author: and the various exceptions, of no unfrequent occurrence, are not unnaturally accounted for. Thus, the consideration of age may yield to some other point of attraction arising out of the physical or moral qualities of the object; and this is especially the case in temporary attachments, where the idea of permanence does not exist.

"Thus, love does not depend on abstract beauty, but on such differences as are consistent with an instinctive feeling of suitableness, which deeply affects us, which first acts upon and agitates the imagination, and which that faculty afterwards acts upon and aggrandizes. Sometimes an accidental, subordinate, and injurious difference, and the association founded upon it, influence this affection; and, by a strange blunder, the mere accidental circumstance, in after-life, is substituted for that with which it was associated." (p. 125.) . . . Hence "we frequently see women, in spite of ugliness and the absence of other combinations, attract and engage in marriage men who might have commanded beauty, accomplishment, and fortune. Certain it is (Mr. Walker adds) that love, thus excited by differences, is favorable to fecundity;" while it has been observed, on the other hand, "that, if persons of similar temperament are joined together, this similitude both produces a series of quarrels and becomes a remarkable cause of sterility.* The beneficial tendency of this law of difference does not terminate here: it leads to those slight crosses in intermarriage between persons of different organization which are as essential to the improvement of the races of men as we have found them to be to those of animals." (p. 125.)

In the section on Marriage, the author has again most unnecessarily gone out of his way, to examine the question whether violation of an adult female by a single man be possible under ordinary circumstances, and whether conception can follow such intercourse. We are disposed to agree with him in thinking that medical jurists have attached rather too little weight to the influence of the horror and disgust which must, we should think, prevail in the mind of a virtuous woman at such a time, on the acts of the organic system which are necessary to render such a coitus effectual. He fully admits the involuntary character of these acts, and allows that they may occur during a state of insensibility; but, considering the influence of violent mental emotions on other involuntary functions,—as, for example, the secretion of saliva, gastric juice, bile, &c.—he questions, and we think reasonably, the possibility of impregnation where the adverse feelings are strongly excited. It is another point to be considered, however, which has been overlooked by our

* It has been very elegantly observed (by a German author, we believe,) that love does not exist between souls that are *in unison*, but between those that *harmonize*. Only our musical readers can appreciate the force of this remark.—Rev.

author, whether a woman who has resisted to her utmost ability may not, when at last overcome, be incapable of resisting the ordinary sexual feelings excited by the coitus, and thus conceive altogether against her will. We do not see how this is to be decided, since, as Mr. Walker justly observes, little credence is to be given to the assertions of women upon these points; but, in the practice of our courts at present, so much attention is very properly given to collateral circumstances, in accusations of this kind, that the fact of conception, or the reverse, is not held to be of much importance.

In the next section, "on the Propagation of Forms and Qualities," our author introduces his particular views by a quotation from Pliny respecting the usual dissimilarity of children to one or both of their parents, upon which he observes,

> "This assertion is more worthy of Pliny than of Camper; its latter part is entirely untrue. I will venture to say, that there never was a child that did not strikingly resemble both its real parents, if resemblance was looked for where it ought to be; as I shall point out in the sequel. But such assertions show the actual state of knowledge on this subject." (p. 138.)

Several well-known facts are then quoted to show the tendency to propagation of varieties of stature, form, &c., as in the case of the six-fingered family, the porcupine family, the gipsy and Jewish races, &c.; and, in the following section, the author enunciates his newly discovered laws regulating the resemblance of progeny to parents. These we shall give in his own words, forewarning our readers, however, that they are partly based upon Mr. Walker's peculiar theory of the functions of the cerebellum, (or *cerebel*, as he somewhat affectedly terms it,) which he regards as the organ of the will, and as therefore especially connected with the locomotive system. We need not stop to point out to our readers how this hypothesis of the uses of the cerebellum is at variance with the doctrines of phrenology; as, on the present occasion, our object is merely to give an account of Mr. Walker's opinions and views.

> "Each parent communicates a distinct series of organs; and the only modifications which the organs communicated by either parent undergo are chiefly, if not altogether, such as are necessary to harmony of action with those communicated by the other parent, and such as are produced by difference of sex. Where both parents are of the same variety, one communicates the anterior part of the head, the osseous or bony part of the face, the forms of the organs of sense, (the external ear, under lip, lower part of the nose, and eyebrows being often modified,) and the whole of the internal nutritive system, (the contents of the trunk, and consequently its form, in so far as that depends upon its contents.) The resemblance to that parent is consequently found in the forehead and the bony parts of the face, as well as in the shape of the organs of sense, and the tone of the voice. The other parent communicates the posterior part of the head, the cerebel situated within the skull immediately above its junction with the back of the neck, and the whole of the locomotive system, (the bones, ligaments, and muscles.) The resemblance to that parent is consequently found in the backhead, the few more moveable parts of the face, and the external forms of the body, in so far as they depend on the muscles, as well as the form of the limbs, even to the fingers, toes, nails, &c." (p. 150.)

As popular illustrations of the truth of these positions, the portrait of Queen Victoria is compared with those of her father and mother; from which it appears that she has the more capacious forehead of the latter, with the general features, especially the nose and mouth, of the former:

and, on the other hand, the Duke of Reichstadt is shown to have possessed the high forehead of his father, Napoleon, and the wide backhead and developed lips of his mother, Maria Louisa. That such partial resemblances are of no unfrequent occurrence, every one must be aware; but that they are constant will, we suspect, be found rather difficult of proof. The chief point, however, which Mr. Walker seeks to establish is the association of the development of the different systems with that of the parts of the head and face, as already described. In support of this position, he brings forward a valuable body of testimony from the observations of Mr. Knight and others, communicated to him: these are, however, deficient in one respect, as they do not indicate the connexion between the cerebellum and locomotive system. Mr. Walker gets over this difficulty by remarking that, in quadrupeds, (upon which these observations have chiefly been made,) the form of the cerebellum cannot be traced externally, owing to the attachment of the powerful muscles and ligaments of the occiput. Strong in his own theory, he says,

"Concealed, however, though the backhead is in these animals, we have proofs of its various developments in the various developments of the muscular system, with which the former must always correspond, and which at all events show what each parent communicates." (p. 162.)

This is a most complete *petitio principii*, as every reader must admit. The very point which remains for Mr. Walker to prove is, that not only a certain form of face and forehead, which may be derived from either parent, coincides with a certain development of the nutritive system, but that the other parent always communicates the cerebellum and locomotive system, of which no satisfactory evidence is given in the work before us. However, we are not prepared to deny the truth of his doctrines, which can only be established or refuted by observation; and for directions as to their verification, with which we have no fault to find, we may refer to the work itself. The characters of two families are closely examined, by way of scientific illustration; and the modifying influence of particular organs upon each other is pointed out. No physiologist who reflects upon the frequent modifications of this kind which are presented to him in the different classes of animals, in order to adapt a particular function to the general conditions of existence, can refuse to admit a subordinate agency of this kind, if the truth of the presiding laws be established.

The principle that either parent may give either series of organs is remarkably illustrated in the history of the *ancon* breed of sheep, which also exhibits the tendency to the perpetuation of accidental varieties, under particular circumstances. This we shall detail, with some additional circumstances not mentioned by Mr. Walker. In the year 1791, one of the ewes on the farm of Seth Wright, in the state of Massachusetts, produced a male lamb, which, from the singular length of its body and the shortness of its legs, received the name of the *otter* breed. From the curvature of its forelegs, which caused them to appear like elbows when the animal was walking, Dr. Shuttack called it *ancon* (from *αγκων*.) This physical conformation, incapacitating the animal from leaping fences, appeared to the neighbouring farmers so desirable, that they wished it continued. Wright determined on breeding from this ram, and the first year obtained only two, with the same peculiarities. The following years he obtained greater numbers; and, when they became capable of breeding with one another, a new and strongly marked variety, before unknown to the world, was established.*

"When both parents are of the otter or ancon breed, their descendents inherit their peculiar appearance and proportions of form. I have heard but of one questionable case of a contrary nature. When an ancon ewe is impregnated by a common ram, the increase resembles wholly either the ewe or the ram. [These observations evidently apply to shape alone, especially to the peculiarity under consideration.] The increase of a common ewe, impregnated by an ancon ram, also follows entirely the one or the other. Frequent instances have happened where common ewes have had twins by ancon rams; when one exhibited the complete marks and features of the ewe, the other of the ram. The contrast has been rendered singularly striking when one short-legged and one long-legged ram, produced at a birth, have been seen sucking the dam at the same time." (p. 164.)

Upon this principle Mr. Walker subsequently explains the alleged cases of superfœtation, in which twins of different colours have been produced: more facts are, however, required to establish one doctrine or the other.

With regard to the communication of the psychical peculiarities of the parents, it results, from Mr. W.'s theory of the functions of the brain, that one should give the organs of sensation and observation, situated in the anterior portion of the cranium, and the other those of passion and volition, which occupy the posterior portion, while the intermediate middle part is divided. In our present ignorance on those matters, it need scarcely be observed that this must be regarded as merely hypothetical. Much, however, may be learned by careful and unprejudiced observation; and we set a high value on such facts as those communicated by Mr. Knight, in the section on the Hereditary Transmission of Mental Faculties, as well as in his paper on the same subject in the Philosophical Transactions for 1837. They unequivocally prove that *acquired* habits (and we must suppose, therefore, the peculiarities of organization which are connected with these habits,) may be transmitted to the offspring so as to be manifested in them without any tuition. As regards the lower animals, however, it seems to us that these facts harmonize with the general principle laid down by Mr. Lyell, that the instincts thus propagated have a relation to those natural to the species, being either necessary to its preservation in a wild state, or, in the case of the dog, to its peculiar association with man. The influence of the education of successive generations of the human race on the character of the progeny, is a question of immense importance; and we agree with Mr. Walker and Mr. Knight that sufficient attention has not been paid to it.

But the question naturally arises why certain minute differences should exist between children who present the same general resemblances to their parents. To a question of this kind put to him by a lady, Mr. Walker could only make the following reply, being prevented by "regard to propriety" from giving the more explicit answer, which he subjoins:

"'*Observe that all these differences in features are mere modifications of your own, such modifications as you yourself might assume under the influence of different emotions, such modifications as you actually have assumed, and therefore have, in these very instances, communicated.*'

* Philosophical Transactions, 1813.

"To explain this most important and interesting point more methodically and in detail. The reader has seen that organization and function are communicated from parents to progeny; he knows that each distinct organization must produce functions equally distinct; he knows that function always reacts on organization, as is shown by the improved forms which well-directed exercise produces on one hand, and the deteriorations which labour causes on the other; he has seen that the practice of performing certain acts in parents gives a distinct tendency to the performance of these acts in progeny; he knows, in short, that organization and function in the parent, are the real and only causes of organization and function in the child. Can he then doubt that the peculiar state of the organization, and the peculiar exercise of every function at the moment of erotic orgasm, must exert the *most* powerful, the *most* undivided influence over the organization and function of the delicate, susceptible, and plastic *ens*, then and by these very acts called into existence?" (p. 182.)

Most of our readers know that this doctrine is by no means novel; and there will be few indisposed to admit that the *habitual* state of the parents has an important influence on the offspring. But we cannot go so far as Mr. Walker in supposing that upon the mere *transitory* conditions, whether of mind or body, at the moment of conception, depends so much of the subsequent development of the new organism. It must be remembered that the influence of the female is continued through a long period; and there can be no doubt that changes in her bodily and mental state may be to a certain extent communicated to the fœtus during pregnancy. We believe several instances have existed of apparent defects in the organization of children begotten in drunkenness; but we are equally assured that the child in utero may be influenced, even as to features and disposition, by impressions made on the female parent during pregnancy; so as to have a resemblance to a third person; and (to obviate cavil on a delicate point) to a female as well as male relative or acquaintance. Upon the *relative* conditions of the parents during the fertile coitus, Mr. Walker imagines the determination of the systems which each communicates to the child to depend. Thus in whichever parent sensibility is predominant over volition, or predominant to the greatest extent (since both may be in this state), that one communicates the anterior part of the brain with the vital system, and vice versâ. "We can no longer wonder, then" he continues, "that several children, having the organs of sense either of the father or of the mother, should differ as to each of these, and as to every feature, according to the general activity and the particular action of each at the moment of creative power."

The influence of the emotions or imagination of the female parent upon progeny is illustrated by a collection of very curious cases, which we have not space to quote; but that the law regarding the communication of the systems in general is not quite so definite as Mr. W. imagines, seems to us proved by those cases of twins in which, on Mr. W.'s own showing, the vital system is communicated to one child by the father, and to the other by the mother, and the converse as to the locomotive system. Every one must have noticed the close resemblance which twins usually bear to each other; and there is a considerable preponderance in the number of those twin births where both children have been of the same sex; but if Mr. Walker's law of the communication of organization be true, there should be none of those exceptions which he has brought forward apparently in ignorance of their real bearing.

With regard to cross-breeding, or union of parents belonging to different races, Mr. W. states the following law to be founded on observation, "that where the parents are of equal age and vigour, the male gives the backhead and locomotive organs, and the female the face and nutritive organs;" and thus ingeniously accounts for the existence of this tendency, which is not, however, universal, and indeed appears (from facts as well as theory) to be less strong in proportion as the cross is feeble, or the parent stocks approach each other.

"If no being can desire that of which it is already in possession—if, on the contrary, it must desire most that which differs most (if not incompatible) it cannot be wondered that in crosses, where the desired difference is greatest, the male, in whom desire is most ardent, should stamp the systems by which he exercises desire, the voluntary and locomotive, upon the progeny. Mr. Theobald, of Stockwell, an extensive breeder, informs me that he has always thought that strong volition and great ardour on the part of the male stamps his form (*general form* depending, as already explained, chiefly on the *skeleton* which is the *basis of the locomotive system*) on progeny, a direct and singular corroboration of the cause just assigned." (p. 202.)

If, however, there be a decided difference in age, or such an inferiority in the breed of the male parent, that the father is much less vigorous than the mother, the shape is given by the latter. As the reproductive system, a part of the vital, is predominant in the female, it follows that the prolific tendencies of a cross-breed should be greater than those of the original stocks; which is well known to be the fact. But discrimination is necessary in continuing a mixed breed, from the cause just mentioned; for as the progeny of the originally distinct parents *may* have different combinations of their organs, the produce of such would not improbably reform the original conditions of the parents. To avoid this, it would, of course, be only necessary to select such individuals to continue the race as exhibit the correspondence of characters which it is desired to perpetuate; and this plan has been found to answer by skilful breeders, whilst those ignorant of the principle are unable to preserve it. Many curious examples of this kind, communicated to him by uninterested observers, are detailed by Mr. Walker. The general similarity of characters which is required for the purpose of preserving the race is very different from that *quasi* identity which is the principle of close in-and-in breeding; and when any new race is established, and thus carefully propagated, it is soon diversified by the modifications and accidents arising in an enlarging herd or flock, which permit the practice of that *selection* which is essential to the continued vigour of the breed.

A law of an opposite character prevails, according to our author, in strict in-and-in breeding; and to this we subjoin his mode of accounting for it.

"Where both parents are not only of the same variety, but of the same family in its narrowest sense, the female gives always the backhead and locomotive organs, and the male the face and nutritive organs." (p. 226.) "As no being can desire that of which it is already in possession, as in animals bred in-and-in, there is little or no difference, little or nothing to be desired,—as no being can feel sexual excitement towards itself,—as organs unexcited do not act,—it is not to be wondered that, in in-and-in, the male no longer stamps his voluntary and locomotive systems upon the progeny." (p. 229.)

In the same manner the early failure of the reproductive power is to be accounted for, the generative system being derived with the other vital organs from the male in whom it is least predominant. The prejudicial effects of this mode of breeding are so well known in the human species, as well as among domesticated animals, that we shall not enlarge upon them, but simply state that Mr. Walker has collected a valuable body of facts in support of his doctrines. To the breeder of cattle the distinctions between the results of union of different relations will doubtless be interesting and valuable; but as the human physiologist has not many opportunities of observing the offspring of a brother and sister, still less of a grandfather and granddaughter, he will not be able to verify or disprove them.

With regard to the communication of sex, Mr. Walker regards it as depending upon the relative vigour of the parent, and the quantity of the reproductive liquid; so that although the vital system, of which the generative organs are a part, may be given as a whole by one parent, the character of those organs may be determined by the other. Although this doctrine finds support in the common opinion of the preponderance of boys while man is in the most flourishing period of life, and of girls in youth and old age, more accurate statistical examination appears to disprove it, and at the same time to indicate another curious condition on which sex depends. The enquiries of M. Holfacker, in Germany, give as their result that, if the father be younger than the mother, or of the same age with her, the proportion of boys to girls is about 90 : 100. If he be two or three years older, the numbers of the different sexes are, on the average equal; but the proportion of boys goes on increasing with the predominance of age on the side of the father, so that if it amount to eighteen years or more, it is nearly double that of girls, and this is the case when the father is past middle age, as well as when in the vigour of life. These results are, it is true, deduced from a limited number of observations; but they so closely coincide with those of Mr. Sadler, that both must be regarded as generally correct. According to the latter, if the male parent be the younger the proportion of boys to 100 girls is 86; if of equal age, the number of boys is 95; if older, by one to six years, 104; by six to eleven years, 127; by eleven to sixteen, 147; and by sixteen upwards, 163.*

A section is devoted by Mr. Walker to the circumstances modifying the laws of resemblance, on the minutiæ of which we cannot at present dwell. We may, however, notice his explanation of the resemblance often noticed between children and more distant relatives, when none can be detected with their parents.

> "The resemblance of a child to its grandfather or grandmother, or to its uncle or aunt, has in it nothing mysterious; but depends upon one of its parents introducing a tendency to some feature, a thicker or thinner lip, a longer or shorter nose, a darker or lighter eye, which was lost in the parent more immediately connected with those relatives, and which, now again introduced, calls into action modifications of form and function which in that parent were at least rendered subordinate, and consequently obscure by other and more dominating ones." (p. 271.)

Upon the foregoing principles our author also explains, and we think very satisfactorily, the tendency of domesticated races to return to the original specific form, which gave rise to all the varieties. This has been

* Quetelet sur l'Homme; tom. i. p. 53.

especially noticed where the animals have entirely escaped from the influence of man; and they are seen not only to reassume their original form but the instincts peculiar to the wild state, which had in their ancestors given way to the instructions of man. The wild dogs of Cuba and the horses of South America are thus circumstanced.

The Fifth and Sixth Parts of Mr. Walker's work relate to the breeding of animals in accordance with the laws already discussed; the Seventh details the vague methods hitherto employed to affect progeny among mankind. From these, we shall here quote several curious facts which we think are of importance to medical men in a practical point of view; and, in doing so, we shall, for the time, leave the peculiar views of Mr. Walker out of the question. Many of these were observed by the late Mr. Knight, whose sagacity and experience, as we have already taken occasion to observe, render his statements particularly valuable.

All breeders of animals acknowledge that their beauty and strength much depend on their being well fed when young. If this is the case with animals, it is a fair inference that the young of man are not exceptions; and we think experience proves that a full supply of nutritive food conduces much to the future size and health of the child.

"The Turks (says Osmer, as quoted by Mr. Walker,) choose these Arabian horses when young, because, if continued long in the hands of the Arabs, they are *small, stunted*, and *deformed* in shape; whereas, when brought into Turkey, a land of greater plenty than the deserts of Arabia, they acquire a greater perfection both of size and shape. Shall we wonder that his offspring, produced in England, a land of plenty, of whom the greatest care is taken, who is defended from the extremity of heat and cold, whose food is never limited, and whose vessels are filled with the juices of the sweetest herbage—shall we wonder, I say, that his offspring, so brought up, should acquire a more perfect shape and size than his progenitor?" (p. 323.)

The effects of over-stimulating animals are singularly like those arising from the similar treatment of men. The blood-horse is in much the same condition as many of the more self-indulgent among his masters, or (yet more) mistresses. Their stimulating and high feeding, hot rooms, hot clothing, insufficient exercise and constant endeavours to ward off every cause which would tend to blunt their bodily or mental sensibilities, all increase to a morbid extent their sensitiveness; and, although they often give them the power for a short time of undergoing great fatigue in the pursuit of pleasure, diminish their capacity of enduring any long-continued exertion either of mind or of body, or, at least, if they are called upon to continued exertion, to bring about their early decay. It is said of Pitt that he was advised medically, in order to prevent hereditary gout, to drink a pint of wine daily even before he went to college, and that his daily allowance even as a young man was two bottles! Pitt must have worked prodigiously hard whilst he lived, but he died at little more than forty. Such men are in much the condition of blood-horses; but many females in the same rank and even in much lower ranks, are even in a more pitiable condition, as they are subject to the same stimulus without the attendant exertion of mind and body, and suffer in consequence all the manifold evils of "idleness and fulness of bread."

"What enormous expense (says Mr. Knight) has been employed in improving the blood-horse in this country: yet the blood-horse is most certainly a much feebler animal in respect to power of carrying weight, or of sustaining the fatigue of a long race, or any race if the ground be soft and wet, than it was fifty years ago. The breeders have destroyed the constitutional powers of the breed of the animal by excess of stimulation, in over-feeding the young animals through successive generations, and they have looked to the legs of the animal for speed, instead of the constitutional power which gives motion to his legs." (p. 329.)

What can convey a stronger lesson to indolent people than the following fact, quoted by Mr. Walker?

"Mr. Thacker observes, that if a stallion be prevented even by accidental lameness from obtaining exercise, he is sure to be deficient in muscular powers, and to convey that deficiency to his offspring. I knew a horse who broke his leg in running a race when three years old, and who has since been kept for covering mares, not being capable of anything else, or even of travelling for that, but his stock are not promising, though he is exceedingly well bred." (p. 330.)

The probability of the children of highly-fed parents requiring more nutritious food than others whose progenitors have lived on simpler fare, as is shown (by the following observations of Mr. Knight's) to be the case with animals, was brought to our mind by the recollection of an attempt made by a lady of high rank to regulate the diet of her children by the simple fare of the healthy neighbouring cottagers. The consequence was the production of strumous ophthalmia in one fine boy, and in several others various low forms of inflammation.

"Mr. Knight observes, 'The improvers, as they are called, of the Durham cattle, feed very highly; their young animals are kept in a fattened state from their birth; and they have brought to market more perfect animals, at an early age, than any other. But every breed of animals which has, through a few generations (two or three is sufficient), been overfed requires similar feeding; and the extraordinary animals which the Durham breeders have sent to Smithfield have come there, I am sure, deeply insolvent; in other words, they have not nearly repaid the expense of feeding them. The offspring of such animals require and can digest more food than others who have lived upon little.'" (p. 332.) . . . "The children of parents who have, through many generations, been well fed would perish if given no more food than would be sufficient for an Irish or Highland Scots peasant child." (p. 333.)

We are inclined to agree with Mr. Knight that excess of application to acquire accomplishments, and we would add sedentary habits of all sorts, tend to produce the defects he mentions. Certainly the number of young women who are unable to suckle their own children from a deficient secretion of milk is in the present day very large, and if the opinion of most observers can be relied on, it is much larger than it was half a century ago. The probable deterioration of form by such habits would be, with many mothers, a strong argument to convince them of their impropriety.

"I am afraid (says Mr. Knight) that some of the defects of the French women are to be found amongst the superior classes particularly, in this country. The girls are generally much more "flat-busted" than they were sixty years ago. I now see them with different feelings; but I can see forms with the same eyes; and several observant women have noticed the change. Look at the pictures of women a century or a century and a half ago, and the bosoms of the women there represented are not similar to those of modern times. Excess of application to acquire accomplishments, and particularly music, has, I suspect, operated injuriously; and I do not think that such stimulants as tea and coffee have been beneficial." (p. 336.)

There is a popular opinion that fat wet nurses have less milk than those whose bodies are thinner, and cattle breeders agree with it.

"The constitutional disposition to form fat (says Mr. Knight) is certainly hostile to the disposition to give milk. Cows which give little milk often present large udders, which contain much solid matter; and, to inexperienced eyes, a two years old Hereford cow would give a promise of much milk where very little would be given." (p. 338.)

The following remarks on fat, by Mr. Walker, are worthy the attention of the physiologist. They are corroborated in some measure by another fact, that nervous patients are generally thin.

"Fat appears to be the means which nature very extensively employs to lower sensibility by interposition between the skin and the central parts of the nervous system. Fat women, and other animals, accordingly, have not only less sensibility and irritability of the skin, but of the organs of sense generally, eyes usually blue, soft, languid, not brilliant, penetrating, &c. Thinner animals, on the contrary, are more generally those of the south, and have more acute sensibility, and, among women, more brilliant eyes, and large mammæ, themselves organs of exquisite sensation." (p. 338.) . . . "When sheep feed upon luxuriant plains, where little muscular exertion is required, a great accumulation of fat takes place. When, on the contrary, they feed upon the scanty herbage of mountains, where great and incessant muscular exertion is requisite, fattening becomes impossible, and sensibility, which would otherwise be unprotected, obtains an exterior covering of the finest wool." (p. 346.)

We shall conclude these miscellaneous quotations, by extracting a few curious facts relative to the transmission to the offspring of the peculiar habits of the parent. In a voyage up the Missouri, by Clarke and Lewis, one of the company was the son of an Indian woman who had married a Frenchman; and this half Indian acquired the power of tracing animals through the trackless wood to any extent—which his companions could not acquire. The whelps of well-trained dogs are, almost at birth, more fitted for sporting purposes than others. Mr. Knight says that an old schoolmaster told him that he had observed a remarkable difference in the capacities of children for learning, which was connected with the education and aptitudes of their parents; that the children of people accustomed to arithmetic learned figures quicker than those of differently educated persons, while the children of classic scholars more easily learned Latin and Greek; and that, notwithstanding a few striking exceptions, the natural dulness of children born of uneducated persons was proverbial.

"Seventy years ago (says Mr. K.) I heard an old schoolmaster remark, in speaking of my late brother's (Payne Knight) great facility of learning languages, that in fifty years' experience, he had never seen a child of wholly illiterate parentage and ancestry, (such being at that time very abundant) who could learn languages, meaning of course Latin and Greek. Being with a friend, about thirty years ago, shooting grouse upon a Welsh mountain, we were joined by a native of the country, who exhibited, with the manners and character of a buffoon, very great powers of combining ideas, and who possessed a good deal of a kind of irregular and uninstructed wit. I pointed out to my friend the difference between him and the other peasants, and observed that, on enquiry, he would prove to be the son of an educated male parent. It proved, upon enquiring, that he was a gentleman's bastard. Being in my parish church, about ten years ago, a little girl, in repeating her catechism got through her part in less than half the time that her companions did, and without missing or hesitating about a single word. She was wholly unknown to me; but I whispered to Mrs. Knight, 'That girl is a gentleman's natural daughter,' and so she proved to be." (p. 177.)

We give these instances, as they are related in the late Mr. Knight's authority. In reference to the children of ignorant parents being incapable of attaining languages, it must, however, be remembered that in their case all early culture has been probably denied them; and those who know (and who does not?) the importance of an educated mother's early care to the future intellect of the child, will doubt whether these instances are as conclusive as they at first appear. We think, nevertheless, their importance in reference to the question at issue, is considerable, when they are viewed in connexion with such facts as those of well-bred dogs producing young more fitted for sporting purposes than others; as well as with such instances as Mr. Knight gives of the bastards of gentlemen, who have had no early care of an educated mother, proving more intellectual than their legitimate companions.

The Eighth Part of Mr. Walker's Treatise is the winding-up or application of the whole subject, giving directions for "Choice in Intermarriage as prescribed by the natural laws and their modifications." Into the details of this our limits forbid us to enter; and we should do injustice to the very ingenious author were we to satisfy the curiosity of all our readers by giving a full abstract. Here, as elsewhere, however, we must complain of the quantity of extraneous matter introduced. With the following general considerations we shall conclude our extracts.

"Certain it is that families, by intermarriages founded on rational principles, and in conformity with the natural laws so clearly established, as prevailing equally among men and lower animals, may, surely, easily, and quickly (some in their first, others in their second generation) raise themselves, in some at least of their members, from deformity to beautiful organization, from disease to health, and from stupidity to high mental ability. Moreover, if the importance of judicious crossing were seen, among the variously organized tribes composing a nation like the British, these benefits, in moderate degree, would be proportionally extended among the mass of the people." (p. 283.) . . . "It can, indeed, be only passion, venality, or pride, that can prevent man from doing for his own progeny, that which natural and universal laws permit him to do for the progeny of every domesticated animal. The only reply that, under these circumstances of actual or daily demonstration, he can make to the invitation of nature and of science is, that he prefers a blind passion to an enlightened one; brutal indulgence, succeeded by life-long disgust, to exquisite enjoyment and permanent happiness; or money, a mere means of pleasure, at the cost of domestic misery—perhaps of conjugal or filial insanity, to actual pleasure for himself and all around him, as well as the progress of children in intellectual improvement and honorable arts—the sole means of abiding fortune; or rank from which he may look up to those above, who despise and spit upon him because he would vainly overtake them in their idiot struggle for a bubble, and down on those below, who therefore naturally hate him for his insolent assumption.

"To those of higher aspirations than these—to those who seek for the improvement of their race, and for mental advancement both in themselves and their progeny, it cannot be wrong in passing to say that the other functions will diminish in energy as the cerebral functions become more intense. Hence men of the highest intelligence are more liable than others to cerebral affections. There are, therefore, prudent limits even to the best employment of the mind." (p. 285.)

There are few, we imagine, who will not in part coincide with these views; and fewer still, we should think, who would entirely unite in them. Mr. Walker seems to have left out of his calculation that there is in the human species such a thing as love, an emotion of the mind quite distinct from the instinctive propensity which man possesses in common with the lower animals, and at the same time completely involuntary. Now, we can imagine it very possible that just as the most ardent affection may exist between two individuals, whose union a wise-judging physiologist would oppose from the fear of consequences to the offspring or to the parents themselves, so it may be completely absent where that which a *breeder* would regard as the most advantageous contrast existed between them, even though the will excited by reason and judgment might not be wanting on either side. Will Mr. Walker tell us, after enlarging as he has done on the necessity of ardent emotion for the vigour of the offspring, that this will arise merely from that harmony which may exist between the forms of the pair which is most conducive to beauty in the progeny?

We think that our readers will perceive that Mr. Walker has in this volume made a valuable contribution to a most interesting and important department of physiology, by the collection, from authentic sources, of a mass of facts sufficient to afford a glimpse of general laws, if not to serve for their establishment. It would be absurd for us or any one else to pronounce a definite opinion on the truth of these laws, without putting them to the test of extensive observation; but, we must say, that we think the evidence adduced in support of them is sufficiently strong to make us desire to see them put to this trial. We must forewarn those, however, who wish to engage in the pursuit, that they must make themselves well acquainted with the sources of modification which Mr. Walker has pointed out, by the attentive study of his book; and if they gain nothing else from its perusal, they will have the advantage of a valuable collection of facts which may avail much in future enquiries, as well as of being put on the right track for their acquirement. Physiologists have, in too many instances, pursued their enquiries into the laws of nature under the guidance of some preconceived notions which are as likely to lead them wrong as right, and which at any rate narrow the boundaries of their investigation. In one or two points we have been obliged to regard Mr. Walker as prejudiced by his peculiar theories; but in the general range of his researches he has given an example which may justly be imitated by others, deriving information bearing on the subject from whatever source it has been offered to him. In this point of view, therefore, his work is of much value to the scientific physiologist; and we cannot but believe that a body of materials might easily be collected from the sources he has indicated, sufficient for the deduction of general principles of unquestionable supremacy. We quite agree with Dr. Birkbeck in thinking that we are much more likely to arrive at "a definite and tangible result" as to the "effect contributed by each sex in the appointed work of reproduction," by "a comparison of the entire and enlarged being with its producers," than by profound researches into "the intricacies of the ovaria, uterus, or seminal fluid." Although we are far from wishing to depreciate these last, the questions we have been considering certainly appear to us beyond their power to resolve.

